# Lactoferrin as a Versatile Agent in Nanoparticle Applications: From Therapeutics to Agriculture

**DOI:** 10.3390/nano14242018

**Published:** 2024-12-16

**Authors:** Emir Akdaşçi, Furkan Eker, Hatice Duman, Priyanka Singh, Mikhael Bechelany, Sercan Karav

**Affiliations:** 1 Department of Molecular Biology and Genetics, Çanakkale Onsekiz Mart University, Çanakkale 17100, Türkiye; emirakdasci@gmail.com (E.A.); furkan.eker@stu.comu.edu.tr (F.E.); hatice.duman@comu.edu.tr (H.D.); 2 The Novo Nordisk Foundation Center for Biosustainability, Technical University of Denmark, 2800 Kongens Lyngby, Denmark; prisin@biosustain.dtu.dk; 3 Institut Européen des Membranes (IEM), UMR 5635, University Montpellier, École Nationale Supérieure de Chimie de Montpellier (ENSCM), Centre National de la Recherche Scientifique (CNRS), F-34095 Montpellier, France; 4 Functional Materials Group, Gulf University for Science and Technology (GUST), Masjid Al Aqsa Street, Mubarak Al-Abdullah 32093, Kuwait

**Keywords:** lactoferrin, nanoparticles, antimicrobial activity, drug delivery, anticancer, agriculture, neuroprotection, toxicity

## Abstract

Nanoparticles (NPs) have emerged as a potent choice for various applications, from drug delivery to agricultural studies, serving as an alternative and promising methodology for future advancements. They have been widely explored in delivery systems, demonstrating immense promise and high efficiency for the delivery of numerous biomolecules such as proteins and anticancer agents, either solely or modified with other compounds to enhance their capabilities. In addition, the utilization of NPs extends to antimicrobial studies, where they are used to develop novel antibacterial, antifungal, and antiviral formulations with advanced characteristics. Lactoferrin (Lf) is a glycoprotein recognized for its significant multifunctional properties, such as antimicrobial, antioxidant, anti-inflammatory, anticancer, and neuroprotective effects. Its activity has a broad distribution in the human body, with Lf receptors present in multiple regions. Current research shows that Lf is utilized in NP technology as a surface material, encapsulated biomolecule, and even as an NP itself. Due to the abundance of Lf receptors in various regions, Lf can be employed as a surface material in NPs for targeted delivery strategies, particularly in crossing the BBB and targeting specific cancers. Furthermore, Lf can be synthesized in an NP structure, positioning it as a strong candidate in future NP-related applications. In this article, we explore the highlighted and underexplored areas of Lf applications in NPs research.

## 1. Introduction

Lf is a multifunctional iron-binding protein found in mammalian milk and various biological fluids, such as saliva and tears [1]. Lf’s N-terminal and C-terminal lobes bind iron ions, retaining this ability even at low pH (~3.5) [2]. Lf’s molecular structure changes depending on its binding status, found in iron-free (Apo-Lf) and iron-bound (Holo-Lf) forms. Lf’s iron-binding ability is particularly significant because it can retain binding at low pH levels (~3.5) [3]. Thanks to its strong iron-binding characteristic, Lf exhibits immense antioxidant activity by inhibiting the Fenton reaction by reducing the iron ions that are needed for reactive oxygen species (ROS) generation [4]. This feature supports ongoing research into Lf’s potential neuroprotective activity. In addition to its antioxidant and immunomodulatory activities, Lf is considered a potential iron chelator that may help reduce the progression of neurodegenerative diseases [5]. Moreover, several mechanisms have been suggested for Lf’s neuroprotective activity. One of the most discussed is the interaction of Lf with surface receptors, which initiates intracellular pathways that protect dopaminergic cells against apoptosis [6].

Lf initiates its antimicrobial activity by reducing the essential iron needed for bacterial growth [7]. Additionally, it can directly interact with cells with its cation-binding residue in the anionic region of the outer membrane, leading to their destruction [8]. The same activities are also mediated with Lf-derived peptides.

Lf is known for binding to various receptor types, enhancing its multifunctionality. Some of these receptors, such as heparan sulfate proteoglycans (HSPGs), are also targeted by many viruses during the initiation of infection [9]. Leveraging this characteristic, various strategies have been developed to use Lf to disrupt the initiation of viral infection by targeting both viral and host receptors, such as cellular glycosaminoglycan [10]. These strategies include applications against viruses such as SARS-CoV-2 [11], human papillomavirus [12], hepatitis B [13], hepatitis C [14], and dengue virus [15].

Moreover, many studies highlight the significant antifungal activity of both Lf and Lf-derived peptides against both fungal and human fungal pathogens [16]. Compared to antibacterial activity, Lf exhibits iron-independent mechanisms against fungus, directly targeting cell wall proteins [17]. Moreover, the antifungal activity of Lf-derived peptides can vary significantly depending on their peptide chain composition and their source [18]. As an example, Biasibetti et al. demonstrated the significant antifungal activity of lactoferrin-derived peptides (lactoferricin and lactoferrampin) [19]. The study revealed that lactoferrin exhibited higher antimicrobial activity against a bacterial strain and yeast, whereas lactoferrampin demonstrated strong activity against the well-known fungal species *Candida albicans* (*C. albicans*). Most importantly, both peptides demonstrated synergistic activity against all tested strains, significantly enhancing their antimicrobial potency. This demonstrates that Lf-derived peptides differ in their antimicrobial activities and can be used together to achieve higher efficacy where potent antimicrobial action is required. Similar studies have investigated the antifungal activity of these peptides [20,21].

**Figure 1:**
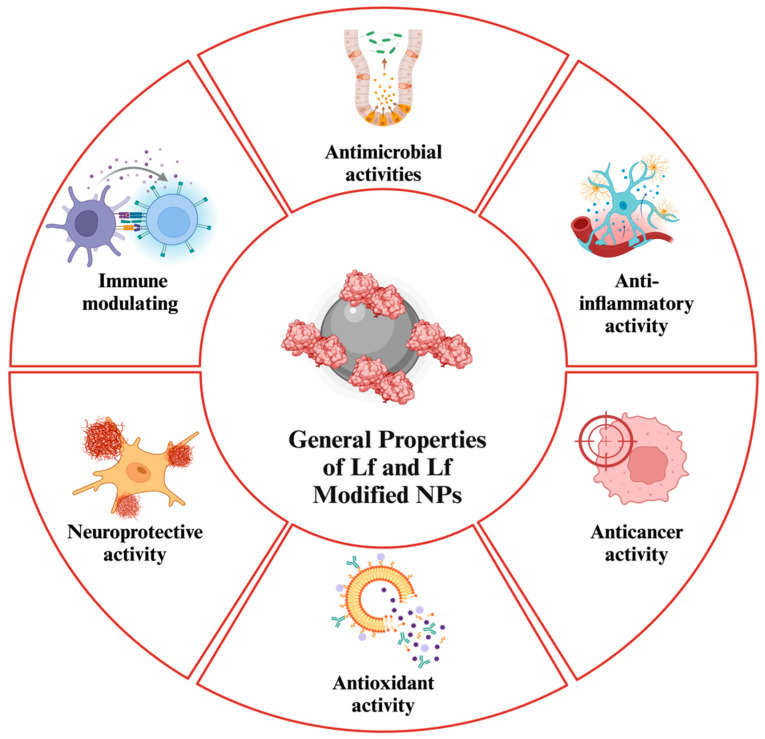
General properties of Lf and Lf-modified NPs [4,7,11].

In addition, Lf is regarded as an excellent immunoregulator and enhancer of host defense, achieved through the modulation of iron homeostasis, stimulation of immune signals, recruitment of immune cells, regulation of proinflammatory secretion, and other mechanisms (Figure 1) [22]. Lf’s immunoregulatory activity is also linked to its anticancer properties. In addition to influencing cytokine levels, Lf can inhibit cellular growth and metastasis of cancer cells, indirectly induce apoptosis by enhancing caspase cleavage, and potentially alter gene expression [23].

Considering these well-known functions, Lf is commonly used in many areas, such as in agriculture to maintain food preservation, dietary supplements to promote gut health and immune modulation, supplements in infant formulas for developing the immune system, and so on [24]. Recently, Lf has been involved in nanotechnology as a supportive molecule and material for NPs to utilize the mentioned functions of the protein [25,26,27].

NPs are structures with small sizes, ranging from 1 to 100 nm, a high surface-area-to-volume ratio, and tunable surface chemistry, and they are classified based on their material composition, such as organic or inorganic [28]. NPs are found in diverse shapes, such as spherical, spheroid, and nanorod, which significantly affect their properties [29,30]. Moreover, the properties of NPs, such as size and shape, are greatly influenced by the chosen synthesis method [31]. Various methods are preferred for NP synthesis, including chemical, physical, and green synthesis, using sources such as bacteria, fungi, algae, and plant extracts [32].

NPs have distinct applications in the current literature depending on their physicochemical properties. Their wide-ranging applications include drug delivery, biomolecule and pathogen detection, photothermal-based cancer cell destruction, bioimaging, biosensor development, chemical catalysis, materials for food packaging and fertilizers, environmental applications, and more [33,34,35].

In this review, we have analyzed the use of Lf in NP technology over the past few years. Lf demonstrates notable potential in NP research as a surface material for targeted delivery strategies. Moreover, Lf can be delivered using various types of NPs for site-specific applications and can also be utilized in NP synthesis to expand the range of applications. Numerous materials are integrated with NPs to enhance therapeutic efficacy and optimize application outcomes. Given its significant potential, Lf is an excellent candidate for integration into NP applications, paving the way for impactful developments and innovative approaches. Considering the substantial potential of both Lf and NP applications in emerging fields, reviews that highlight the current integration of these two areas are essential to drive future innovations.

## 2. Lf Coating and Lf NPs in Delivery Applications

In recent years, Lf has received increasing attention in NP-based drug delivery systems, both as a surface coating and an active NP component (Table 1). Drug delivery applications are one of the most common and highlighted types of applications in NP research [36]. Since Lf is a multifunctional glycoprotein with a high affinity for multiple receptors and diverse surface components, Lf shows significant potential as an agent in nanomedicine.

**Figure 2:**
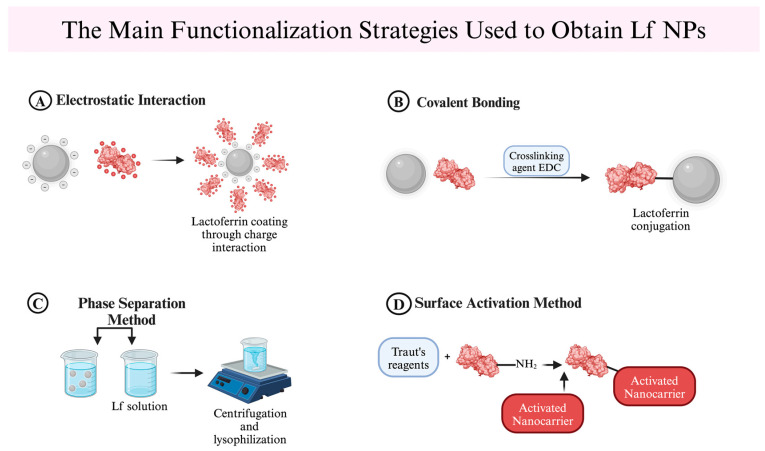
Functionalization methods for obtaining Lf-conjugated NPs for drug delivery strategies [40]. (**A**) Positively charged residues of Lf can adsorb onto negatively charged NPs in an aqueous medium, forming Lf-coated nanocarriers for drug delivery applications. (**B**) Depending on the crosslinking agent used, Lf can be covalently bound to NPs through coupling reactions. Crosslinking agents activate necessary functional groups to enable the formation of covalent bonds between Lf and NP. (**C**) A simpler method involves conjugating Lf to NPs by adding dissolved particles dropwise into an Lf solution. (**D**) Using activating reagents, amine groups on the surface of NPs can be activated, enabling Lf to react with the activated carriers for coating.

Surface modification of NPs with Lf enhances bioavailability and stability while enabling efficient, targeted cellular uptake, which can improve therapeutic outcomes (Figure 2). Furthermore, multiple studies have demonstrated the potential of Lf NPs in drug delivery systems focusing on their synthesis and characterization [37,38,39]. Thus, Lf can initiate its multifunctional properties while ensuring a controlled drug release for the biomolecule. Moreover, some approaches use various types of NPs to deliver Lf itself, aiming to enhance the biological properties of the protein. Still, considering the usage of Lf as a material, this approach has rarely been conducted in the last few years.

**Table 1:** NP-based drug delivery systems with surface modification and Lf NPs.

Application	Study Type	Nanomaterial Property	Main Results	Reference
Deliveries with Lf NPs				
Targeted lung delivery of antibiotic with Lf-included nanocomplex	*In vivo* *In vitro*	Particle size of 237 ± 3.5 nm Spherical morphology Zeta potential of −23 ± 2.2 mV	- Sustained drug release profile. - Minimum inhibitory concentration (MIC) value of 0.5 μg/mL against *Pseudomonas aeruginosa*. - 100% bacterial reduction and zone of inhibition (ZOI) by 29 ± 1.45 mm at the highest concentration (5 μg/mL). - Bacterial reduction in kidneys of infected mice treated with dual drug-loaded PEX, with counts dropping to 3.24 ± 0.067 log_10_ CFU/mL, compared to 18.22 ± 0.194 log_10_ CFU/mL in untreated mice. - Insignificant activity at the lowest concentrations (0.05 and 0.1 μg/mL). - Significant inhibition of bacterial accumulation (6-fold reduction) in kidney and lung tissue of mice. - Reduced oxidative stress in mice, with PEX increasing glutathione (GSH) and catalase (CAT) levels while decreasing malondialdehyde (MDA) levels. - Improved antioxidant parameters and maintained the body weight of mice during the infection. - Improved hemocompatibility with minimal toxicity to hepatic and renal functions	[41]
Delivery of antibiotics and natural compounds with Lf NPs	*In vitro*	Average size of 191.8 ± 11.1 nm Spherical morphology Average zeta potential of +8.03 ± 3.84 mV	- Increased uptake of drug-loaded Lf NPs, up to 90%, by THP-1 cells, surpassing that of free Lf. - Complete inhibition of *Staphylococcus aureus* (*S. aureus*) strain Newman, at concentrations of 25 and 50 μg/mL. - Retained stability of Lf NPs following storage for over 30 days at 4 °C.	[42]
Curcumin-loaded Lf NPs for ulcerative colitis treatment	*In vivo* *In vitro*	Particle size of 229.03 ± 6.20 nm, increasing to 287.54 ± 11.38 nm following folic acid binding Spherical morphology Zeta potential of −10.44. ± 0.19 mV	- Improvement of the loading efficiency of curcumin, up to 95.08%, following incorporation of Lf in the nanosystem. - Increased tight junction protein expression (ZO-1, Occludin, Claudin-1) levels in colon tissues by Lf-included nanosystem, compared to the free curcumin and curcumin-NP groups. - Suppression of TLR4, MyD88, and NF-κB protein levels in colon tissues of UC mice treated with the Lf-included nanosystem. - Restoration of microbial flora diversity, with increased *Bacteroidetes* and decreased *Firmicutes*, following treatment with the Lf-included nanosystem in UC mice.	[43]
Microencapsulated Lf NPs for docetaxel and atorvastatin delivery in the oral treatment of colorectal cancer	*In vivo* *In vitro*	Particle size of 203.6 ± 4.28 nm Spherical core–shell structure Zeta potential of +13.1 ± 1.72 mV	- Effective internalization of drug-loaded Lf NPs by Caco-2 cells along with lower half maximal inhibitory concentration (IC_50_) values compared to free drug samples. - Sustained release of NPs in rat cecal content without degradations observed in the upper gastrointestinal tract. - Suppression of p-AKT, p-ERK1/2, and NF-κB levels and activation of caspase enzymes	[44]
Production of Lf NP encapsulated gold complexes, (Lf-C2), to cross blood–brain barrier (BBB) in glioma treatment	*In vivo* *In vitro*	Average diameter of 58.66 nm Spherical morphology Zeta potential of −13.6 mV	- Successful crossing of the BBB by Lf-C2 NPs compared to C2 alone. - Increased inhibition rates on glioma growth with Lf-C2 NPs (68.6%), compared to the free C2 (21.6%). - Achievement of higher, 88.2%, apoptosis rate in LF-C2 NP-treated tumor tissues compared to 21.2% and 6.9% for C2 and NaCl, respectively.	[45]
Production of zein–glycosylated Lf NPs for improved stability and bioaccessibility of 7,8-dihydroxyflavone (7,8-DHF)	*In vitro*	Size ranging between 78.67 nm and 87.24 nm Zeta potential values between +21.63 mV and +23.45 mV Spherical morphology	- High encapsulation efficiency (above 98.50%) with zein–glycosylated Lf NPs. - Improved bioaccessibility with the existence of Lf, reaching up to a maximum of 84.05%, while free 7,8-DHF achieved only 18.06%. - Increased retention percentage with the addition of Lf, rising from 12.35% to 43.21% under dark conditions at 50 °C. - Enhanced stability over 30 days of storage compared to zein NPs alone.	[46]
Fabrication of zein–Lf NPs for encapsulation of 7,8-DHF	*In vitro*	Average particle size of 74 nm Spherical morphology Zeta potential of +26.93 mV	- Approximately 30 times higher water solubility with zein–Lf NPs (231.60 μg/mL) than that of 7,8-DHF alone (7.12 μg/mL). - Improved bioaccessibility with zein–Lf NPs (63.51%) in comparison to free 7,8-DHF (18.06%) and zein–DHF (31.85%). - Enhanced chemical stability with zein/LF NPs, retaining 27.4% of 7,8-DHF, while free 7,8-DHF was nearly degraded after 15 days at 25 °C under light.	[47]
Development of disulfiram-loaded Lf NPs (DSF-LF-NPs) for the treatment of inflammatory diseases	*In vitro* *In vivo*	Approximate size of 160 nm Spherical morphology Zeta potential around +10 mV	- Protection against LPS-induced sepsis in mice. - Protection against DSS-induced colitis supported by improved disease activity index (DAI), reduced body weight loss, preserved colon length, and minimized epithelial damage and inflammatory cell infiltration. - Reliable safety profile that enables further use.	[48]
NP Modification with Lf				
Anticancer Research				
Production of Lf-coated mesoporous maghemite NPs for the delivery of anticancer drug doxorubicin.	*In vivo* *In vitro*	Diameters around 118 ± 2.86 nm, reaching up to 130 ± 1.48 nm following Lf combination Spherical morphology	- Improved inhibition of cancer cell proliferation and targeted delivery into desired areas. - Enhanced toxicity towards breast cancer cells with Lf-Doxo-MMNPs, supported by IC_50_ value of 20 μg/mL. - Increased tumor growth inhibition (TGI) in mice treated with Lf-Doxo-MMNPs compared to formulations without Lf and Doxo alone. - Increased TNF-α, Fas, Bax, and caspase-3 expression levels with Lf-Doxo-MMNPs at a concentration of 20 μg/mL.	[49]
Synthesis of mesoporous silica NPs, coated with Lf shell, for breast cancer therapy	*In vitro*	Average size of 284.4 nm Spherical morphology Zeta potential of +15.8 mV	- Highest cytotoxicity towards MCF-7 breast cancer cell lines, supported by the lowest combination index (CI) of 0.885 in comparison to free drugs. - Improved cellular uptake of NPs to MCF-7 cells with formulations containing Lf as a targeting ligand.	[50]
Development of Lf-containing nanosystem to mitigate doxorubicin-induced hepatotoxicity	*In vitro* *In vivo*	Particle size reaching up to 268.5 ± 6.4 nm, from 209 ± 3.8 nm, following incorporation of Lf Spherical morphology Zeta potential of −12 ± 1.1 mV	- Prevention of gastric degradation following Lf-included double coating. - Alleviation of doxorubicin-induced hepatotoxic effects along with maintenance of body weight in mouse models.	[51]
Brain Targeted Deliveries				
Lf-functionalized resveratrol-loaded cerium dioxide NPs (LMC-RES) with neuroprotective activity against Alzheimer’s Disease	*In vivo* *In vitro*	Diameters of 90 nm and 120 nm for MC and LMC NPs, respectively Hollow nanosphere morphology Zeta potential of −51.3 ± 2.85 mV for MC and −44.0 ± 2.32 mV for LMC No further information was indicated for LMC-RES	- Successful penetration into BBB, leading to neuronal protection. - Sustained release of resveratrol with high biocompatibility. - Improved drug release rate with LMC-RES, reaching up to 80.9 ± 2.25% after 24 h. - Inhibition of oxidative stress in SH-SY5Y cells through the Nrf-2/HO-1 signaling pathway.	[52]
Development of Lf-modified berberine nanoliposomes (BR-Lf) against Alzheimer’s Disease	*In vivo* *In vitro*	Quasi-circular morphology	- High entrapment efficiency following Lf modification. - Inhibition of acetylcholinesterase (AChE) activity and apoptosis in the hippocampus. - Significant improvement in spontaneous alternation behavior in the BR-Lf group of mice. - Inhibition of tau over-phosphorylation in the cerebral cortex.	[53]
Synthesis of Lf-included polymeric nanocarriers (F-PMBN-Lf) for the delivery of *frankincense* against Alzheimer’s Disease	*In vivo* *In vitro*	Increased particle size of 106.6 nm, from 70 nm, with Lf modification Rod-shaped morphology Zeta potential of −3.8 mV	- Inhibition of scopolamine-induced increases in AChE and GSH. - Sustained release of *frankincense* by the incorporation of Lf, with a release rate of 18.2% after 48 h. - Alleviation of depression and stress by F-PMBN-Lf. - Improvements in short-term memory.	[54]
Other Deliveries				
Lf-decorated nanoconjugates for targeted curcumin delivery	*In vitro*	Depending on the temperature and humidity, sizes ranged from 228.84 ± 11.74 nm to 245.63 nm ± 25.44 nm Same conditions were also applied for zeta potential values, ranging from −30.28 ± 2.11 mV to −32.01 ± 3.21 mV Spherical morphology	- Controlled release of 78.12% of curcumin under acidic conditions (pH 5.8). - Increased anticancer effects through functionalization with Lf, supported by cell viability results ranging from 98.02 ± 1.19% to 94.23 ± 1.45%. - Improvement in bioavailability following Lf coating.	[55]
Development of Lf-modified ternary NPs for the delivery of curcumin	*In vitro*	Average diameter of 144.7 nm Size of 174.7 nm and 205.4 nm, at pH 6 and 7, respectively. Spherical morphology Zeta potential of −28.4 mV	- Increased cellular uptake of curcumin up to 89.5% after 12 h of treatment, compared to sole curcumin treatment at 61.9%. - Improvement in bioaccessibility of curcumin through encapsulation, from 22.1% to 53.6%. - Increased anticancer effects on HT-29 and CT-26 cells in a dose-dependent manner.	[56]
Development of Lf-bearing gold nanocages (AuNCs-Lf) as gene delivery systems against prostate cancer	*In vitro*	Particle size of 105.40 ± 0.43 nm, 103.30 ± 1.31 nm, and 127 ± 1.62 nm, for AuNCs-Lf, polyethylene glycol (PEG)-conjugated AuNCs-Lf and polyethylenimine (PEI)-conjugated AuNCs-Lf, respectively Zeta potential of 19.90 ± 0.45 mV for AuNCs-Lf, 26.70 ± 0.37 mV for PEG-AuNCs-Lf, and 28.70 ± 0.38 mV for PEI-AuNCs-Lf	- Increased gene expression levels with the incorporation of Lf into nanoconjugate, evidenced by a 1.71-fold increase compared to conditions without Lf. - Increased DNA cellular uptake (reaching up to 8.65-fold) compared to naked DNA.	[57]
Lf-decorated nanostructured lipid carriers (NLCs) for leukemia treatment	*In vivo* *In vitro* *Ex vivo*	Average particle size of 81.22 nm in all (30–70 mg/mL) Lf-decorated NLCs. Spherical morphology Higher Lf concentrations led to increased zeta potential values, from −18.60 ± 2.26 mV to +14.90 ± 1.84 mV	- Increased stability of NLCs over 120-day period with negligible changes in particle size - Antileukemic cytotoxicity and induction of apoptosis in K562 cells. - Increased cellular uptake by K562 cells following Lf coating. - Enhanced cytotoxic effects with Lf coating, with an IC_50_ value of 19.81 ± 1.01 μg/mL, compared to uncoated particles, at 35.01 ± 2.23 μg/mL.	[58]
Development of curcumin-loaded Lf nanohydrogels against food stimulants	*In vitro*	Average size of 175.80 ± 56.09 nm Spherical morphology Zeta potential of 23.4 ± 2.05 mV	- Improvement in stability; up to 35 days for storage at 4 °C and 14 days for storage at 25 °C. - Increased release rates of curcumin from Lf nanohydrogels in lipophilic compounds compared to hydrophilic ones. - Successful incorporation into a gelatin matrix without degradation over 7 days of storage.	[59]
Lf Delivery with NPs				
Liposomal-Lf-based eye drops	*In vivo* (clinical trial)	No information was provided	- Reduction in the proportion of potentially pathogenic bacteria, from 36% pre-treatment to 9% post-treatment. - Reliable safety profile with no adverse effects reported. - Higher chance of maintaining the saprophytic flora with eyes treated with Lf.	[60]
Liposomal Lf delivery for dry eye disease	*In vitro* *In vivo*	Average size of 85 nm Spherical morphology Zeta potential of +23 mV	- Maintained stability over 60 days at both 4 °C and 25 °C. - Sustained release of Lf from liposomes, reaching 71.44% over 72 h. - No signs of toxicity against HCE-2 cells, with cell viability remaining above 80%. - Increased aqueous tear secretion in the Lf-treated group, showing a 6.25-fold increase after 5 days compared to baseline and a 4.5-fold increase over the saline-treated group.	[61]
Synthesis of Lf-loaded chitosan NPs to alleviate oxidative damage in rats	*In vivo*	Average size of 336.8 nm Semi-rounded morphology Zeta potential of 47.30 mV	- Suppression of oxidative stress and inflammation after treatment with Lf-containing nanocomplex. - Achieved approximately 58% loading capacity and 88% encapsulation efficiency. - Significant reduction in hepatic MDA and nitric oxide (NO) levels, along with increased GSH and antioxidant enzyme activities (GPx, CAT, GST). - Reduced caspase-3 immunoreactivity in the Lf-treated group, compared to the control group.	[62]
Development of Lf-incorporated mesoporous glass scaffolds to enhance osteoblastic cell cultures	*In vitro*	No information was provided	- High biocompatibility, achieved through the integration of Lf, supporting cell proliferation. - Enhanced biomineralization and osteoblast proliferation following incorporation of Lf. - Increased levels of ALP and Runx2, contributing to osteoblastic differentiation.	[63]

### 2.1. Drug Delivery with Lf NPs

**Figure 3:**
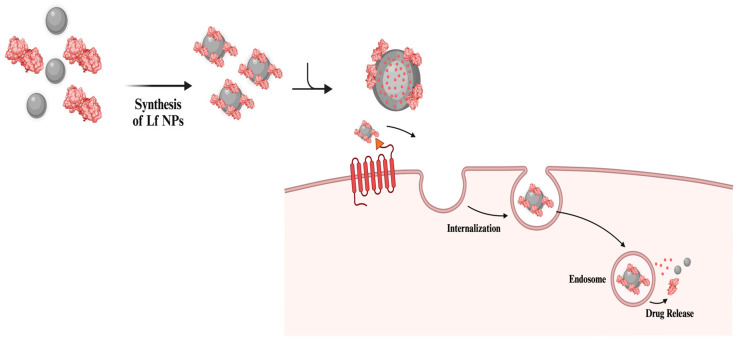
General mechanism of drug delivery of Lf NPs [66].

Protein-based NPs offer high biodegradability and tunable structures that enhance drug loading and enable surface modifications, making various proteins suitable as nanocarriers in drug delivery systems [64]. Due to its multifunctional protein structure, Lf is a strong candidate as a nanocarrier for a variety of drugs (Figure 3) [65].

A recent study used Lf NPs to deliver rifampicin for infection therapy against bacteria [67]. Lf NPs showed encapsulation efficiency by 28.9% with a mean size of 123.6 nm. The *in vitro* antibacterial activity was tested against *Mycobacterium marinum*, *S. aureus*, and *Escherichia coli* (*E. coli*). The results showed that delivery of rifampicin with Lf NPs decreased MIC values (12.5, 8.0, and 0.3125 µg/mL, respectively) by nearly 2-fold compared to solo delivery. A decrease in survival rates of the referred bacteria with Lf NP treatment was directly correlated with the antibacterial activity of Lf. In addition, the Lf NP treatment observed notable membrane disruption and deformation, potentially due to the increased permeability through the NP structure of the protein. While the free antibiotic group reduced biofilm formation by 25%, Lf NP-mediated treatment increased this value to 60%. Moreover, the targeted activity of Lf NPs was demonstrated with enhanced drug accumulation through interaction between Lf and activated macrophages. An *in vivo* mice study revealed that Lf NP-mediated treatment nearly restored all wound areas compared to the control group. The Lf NP group further exhibited reduced fibroblast migration and inflammatory cell infiltration. Finally, Lf NPs reduced bacterium numbers in various organs and blood, thus increasing the survival rate of mice. The mouse model also shows the potential therapeutic activity of Lf NPs against bacterial keratitis for corneal infection.

Another study delivered anti-HIV drugs with sulfonate-modified Lf NPs [68]. Spherical-shaped Lf NPs exhibited 68% drug loading efficiency and 7.2% loading capacity. The cellular internalization and targeting capability of Lf NPs against the HIV-1 viral surface protein gp160 were demonstrated with chitosan encapsulation. While sole treatment of Lf NPs inhibited cell fusion by 20%, drug-loaded particles increased this value to 50%. The direct antiviral activity of unloaded and loaded Lf NPs was shown to be nearly 20% and 90%, respectively.

A study modified Lf NPs for dual-targeting treatment of ulcerative colitis [69]. Coating the Lf NPs prevented undesired interactions in the small intestine, thereby enhancing targeted delivery to inflamed regions of the colon. The results from the *in vivo* experiments showed an improvement in colitis and a reduction in the severity of colonic inflammation symptoms. Additionally, Lf NP-mediated treatment decreased the expression of inflammatory cytokines and triggered anti-inflammatory responses by inhibiting the toll-like receptor 4-linked NF-κB signaling pathway. The NP treatment also protected the intestinal barrier in mice.

Lf is a promising nanostructure for targeted drug delivery systems. Given Lf’s multifunctional properties, Lf NPs can serve as drug carriers in antimicrobial and anti-inflammatory applications. Furthermore, Lf’s inherent ability to perform these activities independently from the carried drug enhances its potential in delivery-based therapeutic applications. Still, when the current literature is analyzed, Lf is commonly used as a therapeutic agent and delivered with other types of nanostructures, rather than being used as a nanocarrier.

### 2.2. Surface Modification of NPs with Lf for Targeted Drug Delivery in Anticancer and Neurological Applications

**Figure 4:**
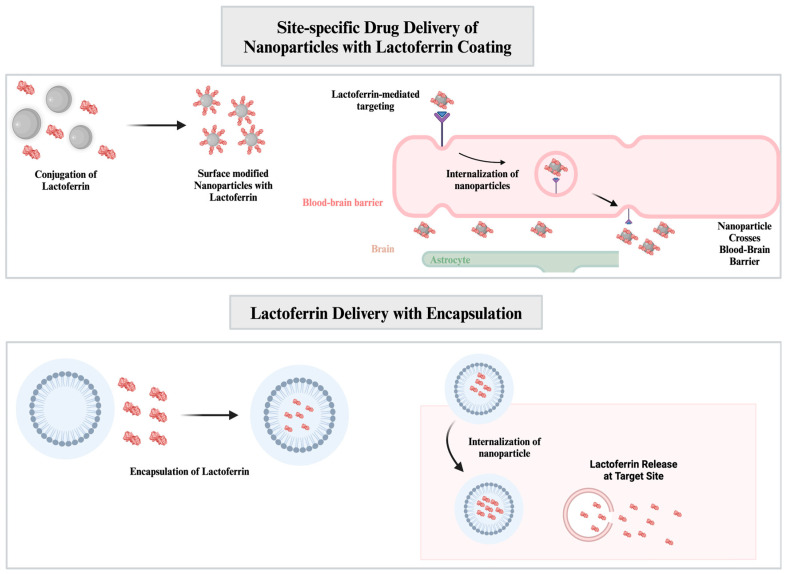
Drug delivery with Lf-conjugated NPs. Lf-conjugated NPs can cross the BBB with receptor-mediated transcytosis, delivering both NPs and encapsulated biomolecules at the target location. Lf can also be delivered with encapsulation into NPs, enhancing its biological activities [71,72].

One of the most preferred approaches for using Lf in drug delivery systems is through modifying the surface of nanocarriers with Lf (Figure 4) [65].

The main strategy is to increase drug internalization through utilizing overexpression of Lf receptors in certain cases. In the current literature, based on the existence of highly expressed Lf receptors, targeted drug deliveries into cancer cells and brain regions commonly use Lf-modified NPs [70].

#### 2.2.1. Anticancer Applications

Nanomedicine, particularly self-assembled nanodrugs, has been a hot topic of study in recent times. This is due to the fast growth of nanotechnology, which has led to the creation of nanomedicine, which has high application prospects in both research and clinical use. Consequently, the key aims are the discovery of novel pharmaceuticals and the optimization of current ones. The development of innovative therapeutics with high efficacy and biosafety is a primary emphasis of cancer therapy, resulting in a change in research towards nanomedicine [73]. Due to the specific benefits that polymer NPs provide, such as longer blood circulation, high drug loading, minimized early leakage, and regulated release of payloads, they have been created for the purpose of providing efficient treatment for tumors to be administered. In the context of synergistic antitumor immunotherapy and conventional medicines, they have shown a significant amount of promise for anticancer treatments [74].

In anticancer applications, NPs are frequently utilized for receptor-mediated targeting of cancer cells, including transferrin receptors, growth factor receptors, and cluster of differentiation (CD) receptors [75]. These receptors play a critical role in the tumor cell cycle or are overexpressed on the cell surface. For example, various NPs are employed for targeted drug delivery against cancer cells via the highly expressed CD44 receptors [76]. The desired receptor-specific targeting can be enhanced with surface modification of NPs. Lf possesses a strong affinity towards low-density lipoprotein receptors (LDLRs), transferrin binding receptors, and CD receptors [65,77,78]. Considering the following examples that are given below, Lf modification can further enhance receptor-specific targeting of NPs in drug delivery strategies.

For example, one study coated poly(lactic-co-glycolic) (PLGA) NPs with Lf and hyaluronic acid for targeting CD44 receptors in lung cancer [79]. Lf-modified PLGA NPs were decorated with polydatin and established sustained release for up to 24 h. An *in vitro* cellular uptake study revealed that Lf coating onto PLGA NPs increased fluorescence intensity from 325.39 ± 11.03% to 1741.13 ± 65.50%, compared to the control group. Moreover, polydatin decoration increased the intensity to the highest level by 3130.66 ± 228.53%.

A similar study also conducted targeted anticancer activity with chondroitin–Lf dual-modified solid lipid NPs (SLNPs) against breast cancer [80]. *In vitro* drug release profiles showed that both dual-modification and only Lf modification led to controlled drug release, compared to unmodified SLNPs. The anticancer activity was determined by using cell line studies (MDA-MB-231 cells). Similar to the previously mentioned study, the authors highlighted that the cell lines were preferred due to their high expression of transferrin, CD44, and LDL receptors. The selective cytotoxicity towards cancer cells was shown during the experiments, whereas only LF modification showed the highest selectivity index, followed by the dual modification. Determining intracellular uptake through fluorescence intensity further supported the selectivity with high values. It was discussed that there is a great possibility that Lf-modified particles demonstrated the highest internalization through receptor-mediated endocytosis. Finally, *in vivo* anticancer studies revealed the following results: the highest reduction in tumor volume compared to the control group (from 1056 ± 93.34% to 274 ± 62.22%), strongest antiangiogenic activity with a 2.33-fold decrease in vascular endothelial growth factor, lowest expression levels of cyclin D1, apoptosis induction, and having the highest area of necrosis.

#### 2.2.2. Targeted Brain Delivery Applications

Another common approach for Lf-modified NPs in drug delivery systems is the targeted delivery into the brain regions. Lf exhibits significant neuroprotective activity against neurodegenerative diseases [4]. It has been proposed that Lf can initiate intracellular activity through receptor binding, such as with heparan sulfate proteoglycans, lipoprotein receptor-related proteins, and other Lf receptors [4,6]. Dopaminergic neurons, which are found to decrease when neurodegeneration is severe, have overly expressed Lf receptors [81]. Moreover, the fact that Lf can initiate receptor-mediated transcytosis through the BBB makes Lf a great choice as a protein for NP modification in drug delivery applications for reducing the progression of neurodegenerative diseases [65].

Although there are only a few recent examples, Lf NPs can still be utilized to induce neuroprotective activity in brain regions. As discussed, Lf exhibits strong neuroprotective activity and can cross the BBB. Therefore, on an NP basis, various types of NPs are modified with Lf for targeted delivery, rather than using Lf as the NP itself or delivering it directly into specific brain regions. A recent example demonstrated the intranasal administration of curcumin–Lf NPs for determining the capacity of neuroprotective effects [82]. The characterized NPs showed ideal properties as drug carriers, with an encapsulation efficiency of 91.2% ± 3.6% and a drug loading of 9.6% ± 0.8%. *In vitro* experiments demonstrated sustained drug release, high cellular penetration, and significant cellular uptake. A PC-12-cell-mediated nerve damage model was employed to determine the protective effect of Lf–curcumin NPs. Although Lf NPs managed to show notable neuroprotective effects when administered alone, their combined application with free curcumin and Lf–curcumin NPs resulted in even higher protective effects, with cell viability improving by over 90%. Furthermore, the NP treatment significantly reduced induced apoptosis and oxidative stress. Most importantly, *in vivo* pharmacokinetic studies revealed that Lf NPs successfully accumulated in brain regions and enhanced the bioavailability of curcumin.

To give a few examples of Lf coating on NPs, an *in vivo* study utilized Lf-functionalized manganese-doped silica hollow mesoporous NPs to deliver resveratrol for treating ischemic stroke [83]. *In vivo* bioimaging studies demonstrated that Lf-modified NPs successfully accumulated in brain regions, exhibiting high fluorescence intensity in neurons and microglia, whereas unmodified NPs were mainly observed in the liver. Additionally, an *in vitro* BBB model demonstrated the significant Lf-mediated crossing of the NPs through the BBB. The modified NPs significantly exhibited two crucial activities: antioxidant and anti-inflammatory effects. Most importantly, a notable reduction in neuronal apoptosis was observed in mice, with a decrease in pro-apoptotic factor expression by up to 64.1% ± 7.83%.

Another similar study modified nanoliposomes with Lf and borneol to mediate targeted delivery of crocetin in mice [84]. *In vitro* tests on HT22 cells demonstrated high cellular uptake and internalization of the NPs. The neuroprotective activity of Lf-modified NPs was determined by an increased cell survival rate of 15–33%, depending on the source of injury. *In vivo* experiments demonstrated the biodistribution of the NPs, highlighting the Lf-mediated brain targeting of liposomes.

The current literature suggests that Lf is an effective agent for surface modification of NPs in targeted drug delivery applications. Lf exhibits a multifunctional protein structure and unique interactions with various receptors, expanding the range of targets in drug delivery. Given the emergence of alternative drug carriers for both anticancer and neurodegenerative applications, Lf could be one of the molecules that precisely fits into NP-based treatments.

### 2.3. Lf and NPs in Delivery Systems for Hepaprotective, Antioxidant, and Anti-Inflammatory Applications

Lf demonstrates both antioxidant and anti-inflammatory activities in several applications, such as in inflammatory bowel diseases [78] and antioxidant biofilms in food packaging [85]. Moreover, oxidative stress and neuroinflammation levels are directly linked to the progression of neurodegenerative diseases [86], and Lf has the potential to reduce these levels significantly [6]. Additionally, several studies have demonstrated the hepatoprotective activity of Lf against induced hepatotoxicity (including NP-induced toxicity) [87,88,89]. As a result, various nanostructures including Lf have been employed with various types of approaches to mitigate the aforementioned toxic effects, including surface modification with Lf, direct delivery of Lf, and incorporation of Lf into nanocomplexes.

An *in vivo* study used selenium NPs to deliver Lf for its hepatic- and immune-modulatory activity for drug-induced hepatic injury [90]. An analysis of biochemicals from mice demonstrated a significant reduction in lipid peroxidation and NO levels, oxidative stress regulation through increased GSH levels, and a reduction in proinflammatory cytokines and apoptotic and pro-fibrotic markers. Moreover, Lf-loaded NP treatment significantly reduced caspase-3 expression in liver tissue nearly to the control levels, lowering the drug-induced hepatotoxicity. The histopathological analysis supported the reduced hepatotoxicity with improved hepatic structures.

A similar *in vivo* study also treated drug-induced hepatotoxicity but through Lf-coated zein NPs as a dual oral carrier [41]. The involvement of Lf as one of the coating materials was thought to increase gastric digestion tolerance, considering the molecular structure of the protein. This was supported by the lowered and sustained drug release of the nanostructure. While observing the distribution of the administered NPs, it was shown that coated NPs exhibited less of an off-target effect, as shown by a low fluorescence intensity in the kidneys and spleen. The oxidative stress parameters were normalized similarly to the previous study, through increased GSH levels and reduced levels of a lipid peroxidation byproduct. Undesired changes in the parameters of both the liver and kidney were changed nearly to the control levels through coated-NP treatment. It is worth mentioning that all of these outcomes were not significant with the solo treatment of compounds or uncoated forms of NPs.

### 2.4. Lf Delivery with NPs

In addition to its use in NP-based delivery systems, Lf’s biological properties are applied in various fields by directly delivering it to NPs. Lf exhibits multifunctional activity in various places [5] and is known for initiating both covalent and non-covalent interactions with various biomolecules [72]. The site-specific delivery of Lf in high efficiency can enhance the therapeutic application of Lf.

#### 2.4.1. Ocular Delivery of Lf with NPs

Lf plays a crucial role in tear fluid due to its multifunctional properties. It is widely expressed in various parts of human eye tissue [91]. Lf shows significant potential as a therapeutic agent for treating dry eye disease and ocular surface infections [92]. Furthermore, ocular surface diseases can significantly reduce Lf concentrations in tear fluid [93], which highlights its potential as a biomarker for dry eye disease [94].

Considering the role of Lf, various delivery systems, including nanostructures as a transporter, have been used for the ocular delivery of Lf. Primarily polymer-based systems, such as PLGA, and lipid-based systems, such as lipid nanocarriers and liposomes, have been used in the topical administration of Lf in ocular delivery applications [95].

For example, a study demonstrated the potential of administering Lf topically to the eye using PLGA NPs in the form of nanospheres and nanocapsules [96]. The nanostructures exhibited efficient, controlled drug release and showed no significant cytotoxicity. An *in vivo* study revealed that the nanostructures could initiate interaction with the ocular surface for up to 5 h. A study showed further evaluation of PLGA-based NPs for delivering Lf, indicating its effectiveness in treating ocular inflammation *in vivo* [97]. Compared to free Lf, NP-carried Lfs exhibited a prolonged and controlled drug release profile in an *in vitro* release study. Ex vivo corneal permeation indicated that Lf’s encapsulation improved penetration speed and administration concentration, resulting in an approximately 1.5-fold higher permeability coefficient and permeated amount than free Lf. The anti-inflammatory and cytotoxicity status of Lf-loaded PLGA NPs were evaluated *in vitro* using HCE-2 cells. Due to the biodegradable nature of the PLGA polymer, the delivery system did not induce cytotoxicity and reduced the expression of cytokines (IL-8 and TNF-α). *In vivo* studies confirmed similar anti-inflammatory activity, with a slight increase in recovery speed and reduction in inflammation, and demonstrated that the treatment passed ocular tolerance tests without irritating.

#### 2.4.2. Lf Delivery for Bone Engineering

Lf exhibits significant potential in the regeneration of bone structures through the induction of proliferation and differentiation of tissues [98]. Despite its potential in bone engineering applications, meta-analyses from recent years highlight the need for further research on Lf in *in vivo* and clinical studies [99]. Although limited, some studies have investigated NP-mediated Lf delivery for bone formation applications.

For instance, an *in vitro* study used alginate chitosan NPs to deliver bovine Lf, aiming to perform detailed characterization and enhance the growth of the MG-63 cell line [100]. The study primarily focused on NP characterization and analyzed changes following Lf loading. The NPs exhibited an encapsulation efficiency of 89.94% and showed the highest interaction rate with Lf at pH 8, with no structural changes after Lf loading. MTT tests demonstrated that Lf delivery increased cell viability by 8.5-fold.

Noh et al. developed Lf-anchored silica NPs for enhancing osteo-differentiation for bone healing and regeneration applications [101]. Lf release patterns exhibited sustained release profiles for up to 28 days. Osteo-differentiation marker changes were analyzed. An increase in alkaline phosphatase was first observed on day 3, with a significant difference noted by day 9. By day 14, the concentration of accumulated calcium levels showed a similar increase. The most significant change was observed in the mRNA expression of osteocalcin and osteopontin, with up to a 3-fold increase.

The same research group used an *in vivo* rat model to demonstrate the induced bone fusion with the developed Lf-carrier NPs [102]. The following results that indicate the enhanced bone fusion in rats were obtained: increased frequency of blood vessels (2-fold increase), a significant increase in osteocalcin concentrations (up to 3-fold increase), and increased mRNA expression levels of osteocalcin and osteopontin (nearly 4- and 3-fold, respectively).

## 3. Antimicrobial Applications of Lf NPs

As a vital component of milk, Lf is an iron-binding glycoprotein that is essential to the innate immune response. Iron homeostasis, immunological response, antioxidant, anticancer, and anti-inflammatory qualities are among its physiological roles; its antimicrobial action has been the subject of the most research. Lf demonstrates efficacy against a range of bacteria, fungi, viruses, and protozoa by sequestering iron, an essential resource for pathogens, while its structural integrity is vital for preserving its bioactivity during processing [103].

### 3.1. Antibacterial

As an initial line of defense, many species generate antimicrobial proteins and peptides. For animals, including humans, milk is an essential source of nourishment. It is also a rich supply of proteins, including Lf, a multipurpose protein that has antimicrobial properties due to its capacity to bind iron. By targeting bacterial pathogenicity mechanisms, iron sequestration, membrane instability, and host cell invasion tactics, Lf has antibacterial effectiveness against a variety of bacterial pathogens [104]. Its broad-spectrum antibacterial inhibitory action has been demonstrated against both Gram-positive and Gram-negative bacteria, successfully preventing the development of *Salmonella typhi* (*S. typhi*), *Streptococcus*, *Legionella pneumophila*, *S. aureus*, and *E. coli* [105].

The physicochemical characteristics of bovine Lf and silver–Lf complexes, resulting from the technique of immobilizing silver onto and/or inside LTF, and the antibacterial activity of these complexes were examined by Pomastowski et al. The antibacterial activity study’s findings prospectively imply that synthesized silver–Lf may find application as a novel, commercially and ecologically viable antimicrobial agent in the food and medical industries [106]. Similarly, Alhadide et al. have used NPs with whey protein films to manufacture Lf packed with iron oxide Fe_2_O_3_-NPs (IONPs) utilizing CONCARPUS extraction, which includes complete three-dimensional structural stability. Using the well diffusion approach, the antibacterial activity of the generated NPs in conjunction with the created whey protein films was examined against *Salmonella enterica*, *S. aureus*, *S. galactiae*, and *E. coli*. As a consequence, they discovered that Lf nanovesicles in whey films produced under optimal conditions were also successfully employed in antibacterial research facilities [107].

A novel strategy for combating pathogenic-resistant bacteria is provided by hybrid NPs, which are made to have various antibacterial activity mechanisms. Silver NPs were created by Abdalla et al. and have antibacterial and anti-biofilm qualities. The silver NPs were surface-functionalized with either GO (AgGO) or LTF (Ag-LTF) after being produced using chitosan and mushroom waste. A surface plasmon resonance band was observed at 430 nm for the silver NPs, whereas AgGO and Ag-LTF exhibited absorption at 402 and 441 nm, respectively. Gram-positive and Gram-negative bacteria were suppressed by silver NPs, Ag-LTF, and AgGO with similar antibacterial efficacy. Ag-LTF did not impact cell survival or migration rate, indicating non-toxicity; however, AgGO and Ag-LTF showed synergistic efficacy against *P. aeruginosa*. According to these results, silver NPs, Ag-LTF, and AgGO could be useful antibacterial agents in the future [108].

### 3.2. Antiviral

Numerous naturally occurring antimicrobial proteins and peptides have shown promise in preventing viral infection, preventing the virus from entering the host cell or influencing the virus at a later stage of development [109]. Lf is a glycoprotein found in external secretions, with many roles, such as antiviral properties and the enhancement of immune response [110].

**Figure 5:**
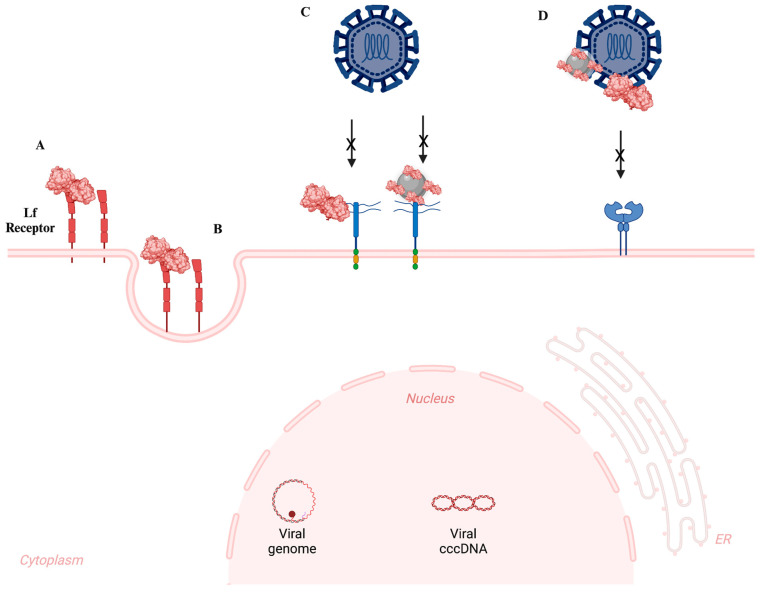
Schematic illustration depicting the potential pathways of antiviral activity by Lf and Lf-modified NPs. (**A**) Lf can be swallowed by endocytosis; the interaction of Lf-modified NPs needs validation. (**B**) The endocytosis of Lf-modified NPs needs validation. (**C**) Lf or Lf-modified NPs can inhibit HSV-1/2 infection by binding to heparan sulfate moieties found in the glycoproteins of the cell surface and extracellular matrix. (**D**) Lf or Lf-modified NPs can obstruct viral binding by attaching to the virus attachment ligands (VALs), thus preventing the virus from connecting to its particular receptor(s) [115].

Within the concept of antiviral mechanisms of Lf, the cell surface and extracellular matrix include heparan sulfate proteoglycans (HSPGs), which Lf can bind to. On the surface of the majority of eukaryotic cell types, these proteins are expressed and consist of a core protein with GAG chains. The negatively charged sulfated groups of heparan sulfate (HS) present in the glycocalyx of the cell surface and viral attachment ligands (VALs) are what bind viruses to HSPGs. Certain viruses can enter cells more easily when HS helps them make contact with the cell surface and fuse with the host membrane [111,112]. Lf may disrupt HSPGs on the cell surface, directly attach to viruses or their receptors, or enhance the antiviral response of the immune system. Nevertheless, several scientists suggest that its potential mechanism of action involves the disruption of the endocytic route of viral infection (Figure 5) [112].

Hepatitis C virus (HCV), herpes virus, respiratory syncytial virus, rotavirus, and HIV are among the viruses against which LF has demonstrated antiviral efficacy [113]. By preventing viral attachment and penetration into the host, Lf has an anti-HSV impact [114]. Both HSV-1 and HSV-2 have been shown to be susceptible to Lf’s antiviral action, although the precise antiviral process varies slightly because of the two viruses’ differing initial attachment to target cells [109,110].

Proteins and NPs can interact to create the so-called interfacial protein corona, which gives the particles particular biological activity. Van der Waals interactions and hydrogen bonds allow bovine Lf to be absorbed into the silver NP surface without changing the stability and shape of the protein, as shown by the work of Nayak et al. [116]. Additionally, bovine Lf (bLf) reduced the cytotoxicity mediated by silver NPs [116]. By directly inhibiting viral attachment, penetration, and infection, Krzyzowska et al. showed that pre-treatment with human Lf-functionalized gold and silver NPs sized 10 or 30 nm reduced HSV-2 infection. According to the study, Lf bonds to HSPGs on the cell surface more efficiently in nanometal conjugates, forming a barrier that prevents HSV-2. Lf conjugates had an extra immunomodulatory impact during vaginal infection; however, all tested Lf conjugates reduced HSV-2 titers in *in vivo* tests [117]. In a different study, as an HIV-microbicide, Yeruva et al. developed Lf NPs (TCNPs) loaded with tenofovir and curcumin. HIV-1 replication was successfully suppressed by TCNPs, which had a diameter of 74.31 ± 2.56 nm and an IC_50_ of 1.75 μM for curcumin and 2.8 μM for tenofovir. They demonstrated spermicidal action, produced low cytotoxicity and inflammation in the vaginal epithelium, and offered drug release for up to 8–12 h, making them a promising anti-HIV microbicidal agent [118].

Zidovudine + efavirenz + lamivudine-loaded Lf NPs (FLART-NPs) were produced and assessed by Kumar et al. for their physicochemical characteristics, bioactivity, and pharmacokinetic profile in order to improve the efficacy, bioavailability, and decrease the toxicity of the first-line highly active antiretroviral regimen. Thus, three anti-HIV medications have been effectively encapsulated in NPs by researchers, producing a stable, evenly distributed colloidal solution. The regulated and prolonged intracellular release of medications by the NPs improves their pharmacokinetics without endangering vital organs [119].

A recent investigation posited a synergistic antiviral effect of Zn-NPs coated with LF protein against SARS-CoV-2 infection and the resultant pulmonary fibrosis. This application innovatively enhances cellular absorption and maximizes the antiviral and anti-inflammatory effects of LF by the saturation of LF with biosynthesized Zn-NPs. The superior effectiveness of biosynthesized Zn-NPs and LF-coated Zn-NP nanocomplexes may serve as a potential treatment for SARS-CoV-2 and its associated problems [120].

### 3.3. Antifungal

The escalating prevalence of serious fungal infections, mostly attributed to a growing population of immunocompromised individuals, has rendered the identification of novel compounds with potent antifungal properties, or those that enhance the efficacy of existing antifungal medicines, increasingly imperative. Milk proteins and other natural products are a major source of new antifungal agents [16].

Numerous studies in the literature have demonstrated the promise of Lf, a multifunctional protein, as a broad-spectrum natural antifungal agent due to its strong antifungal activity against a variety of fungi (Table 2). The antifungal efficacy of Lf (both human and bovine sources) arises from its capacity to sequester iron, depriving *Candida* species of this essential nutrient, which results in membrane breakdown and leakage through iron-independent mechanisms [121].

Antimicrobial materials have gained significance for localized therapy to avert microbial resistance caused by systemic antibiotic treatments. The fabrication of electrospun poly(lactic acid) nanofiber membranes infused with bovine Lf at concentrations up to 20% was performed by Machado et al. The resulting fibers are characterized by a smooth, defect-free morphology, with average diameters ranging from 717 ± 197 nm to 495 ± 127 nm, and an overall porosity of around 80%. The synthesized membranes exhibit antifungal properties against *Aspergillus nidulans* by obstructing spore germination and mycelial proliferation [103].

Plasmonic magnetoliposomes (PMLs) are advantageous nanocarriers for simultaneous hyperthermia and localized chemotherapy, owing to their integration of magnetic and gold NPs, which facilitates targeted drug delivery. From this perspective, gold NPs (5–7.5 nm size) and manganese ferrite NPs (28 nm size) functionalized with 11-mercaptoundecanoic acid or octadecanethiol were added to PMLs and loaded with bLf. Using fluorescence microscopy and colony-forming unit counts, the antifungal potential of bLf-loaded PMLs and their internalization process were evaluated in *Saccharomyces cerevisiae*. Thus, PMLs containing bLf exhibit cytotoxicity efficacy comparable to that of free bLf, suggesting that they may be used to deliver bLf in antifungal therapeutic treatments [122].

**Table 2:** Antimicrobial applications of Lf NPs and Lf-incorporated nanomaterial systems.

Antimicrobial Activity of Lf with NPs	Study Type	Main Results	Reference
Antibacterial			
Development of silver–Lf NP-incorporated hydrogels	*In vitro*	- Silver–Lf NPs demonstrated antibacterial activity against both Gram-positive (*S. aureus*) and Gram-negative (*E. coli* and *P. aeruginosa*) bacteria. - Largest zones of inhibition were 11.3 ± 7.5 mm, 10.3 ± 1.5 mm, and 7.3 ± 0.6 mm for *E. coli*, *S. aureus*, and *P. aeruginosa*, respectively, at an NP concentration of 125 µg/mL.	[123]
Development of antibiotic-loaded Lf NPs	*In vitro* *In vivo*	- NPs demonstrated bactericidal activity against *E. coli*, *Mycobacterium marinum* (MM), and methicillin-resistant *S. aureus* (MRSA). - Superior antibacterial effects, in comparison to free antibiotic and antibiotic-loaded bovine serum albumin NPs, were observed. - MIC values were determined as 12.5 µg/mL for *E. coli*, 0.3125 µg/mL for MM, and 8.0 µg/mL for MRSA. - *In vivo* assays on a mouse model highlighted that antibiotic-loaded Lf NPs can promote wound healing by improving intracellular bacteria elimination.	[67]
Synthesis of Lf-functionalized gold NPs	*In vitro* *In vivo*	- Antibacterial activity against both non-pathogenic bacteria, such as *Bacillus subtilis* (*B. subtilis*) and *E. coli,* and pathogenic strains including *S. aureus*, *Enterococcus faecalis* (*E. faecalis*), and *S. typhi.* - Enhanced antibacterial effects, through functionalization with Lf, compared to NPs alone. - Lowest MIC values were determined as 10 µg/mL and 15 µg/mL for *B. subtilis* and *E. faecalis*, respectively. - Largest zones of inhibitions were observed as 8.35 mm for *E. faecalis* and 8.45 mm for *B. subtilis.* - Increased biocompatibility and hemocompatibility in Wistar rats, following incorporation of Lf-functionalized gold NPs.	[124]
Development of Lf-functionalized silver NP-incorporated gelatin hydrogels	*In vitro*	- Dose-dependent antibacterial activity of Lf–silver NPs against *S. aureus* and *P. aeruginosa.* - Increased inhibition zones, from 10.7 ± 3.6 to 12.7 ± 2.3 for *S. aureus* and 10.8 ± 1.4 to 11.9 ± 3.2 for *P. aeruginosa*, when Lf–silver NP concentration in hydrogels was increased from 62.5 μg/mL to 125 μg/mL.	[125]
Preservation of strawberry samples through antibacterial Lf NPs	*In vitro*	- Significant antibacterial activity against *S. aureus*, 0.3 mg/mL MIC value. - Lf NP coating on strawberries with carboxymethylcellulose reduced weight loss from 85% to 60% at day 6. - Significant reduction in counted aerobic mesophilic bacteria. - Reduced physiological changes of strawberries during storage.	[126]
Lf-included nanocomposite for packaging	*In vitro*	- High antioxidant activity by 67.6 ± 1.4% DPPH radical scavenging. - Significant antibacterial activity against *E. coli* and *S. aureus* with 18.5 mm ZOI. - Increased decomposition.	[127]
Antiviral			
Development of Zn-NPs coated with bLf using green synthesis	*In vitro*	- LF-Zn-NPs contained larger particles that measured up to 98 ± 6.40 nm, whereas the biosynthesized Zn-NPs were white, oval to spherical in form, and had an average size of 77 ± 5.50 nm. - The negatively charged surfaces of the biosynthesized Zn-NPs and LF-Zn-NPs were found to have zeta potentials of −20.25 ± 0.35 and −44.3 ± 3.25 mV, respectively. - By attaching to the ACE2-receptor and spike protein receptor binding domain with IC_50_ value of 59.66 μg/mL. LF-Zn-NPs showed a notable *in vitro* delay of SARS-CoV-2 entrance to host cells.	[120]
Development of zidovudine + efavirenz + lamivudine-loaded Lf NPs (FLART-NPs) against HIV therapy	*In vivo* *In vitro*	- Encapsulation efficiency, cellular localization, release kinetics, safety analysis, biodistribution, and pharmacokinetics have all been investigated *in vitro* and *in vivo*. - For each medication, FLART-NPs were produced with an encapsulation effectiveness of >58% and a mean diameter of 67 nm (FE-SEM). - With little burst release, low erythrocyte damage, and enhanced anti-HIV effectiveness in *in vitro* experiments, FLART-NPs deliver the maximal payload at pH5.	[119]
Development of coencapsulated Lf NPs with tenofovir and curcumin to enhance vaginal protection against HIV-1 infection	*In vitro* *In vivo*	- Efficient inhibition of HIV-1 replication, supported with IC_50_ values of 1.75 μM and 2.8 μM for curcumin- and tenofovir-loaded Lf NPs (TCNPs), respectively. - Increased bioavailability and reduced systemic toxicity in rats treated with TCNPs	[118]
Development of Lf-coated Zn NPs against SARS-CoV-2	*In vitro* *In vivo*	- Synthesis of spherical Lf-coated Zn NPs with an average size of 230.8 nm and a zeta potential of −36.85 mV. - Prevention of viral entry through the inhibition of SARS-CoV-2 receptor ACE2 by the NPs. - Effective neutralization of viral particles with an IC_50_ of 16.84 μg/mL, along with inhibition of SARS-CoV-2 propagation in infected cells demonstrating an IC_50_ of 13.56 μg/mL. - *In vivo* efficiency in rat model evidenced by alleviated Bleomycin-induced pulmonary fibrosis through reduced oxidative stress and modulation of overexpressed inflammatory and cytokine responses.	[128]
Development of Lf-encapsulated nanoliposomes against SARS-CoV-2 and Human coronavirus 229E (HCoV229E)	*In vitro*	- Encapsulation of blf into nanoliposomes with sizes ranging between 80 nm and 150 nm. - Enhanced virucidal activity by liposomal bLf (LL), resulting in approximately 80% reduction in infection rates for both tested cell lines. - Maintenance of virucidal activity even at low doses, supported by more than 50% reduction in infection by LL at concentrations below 10^−3^ (%*w*/*v*).	[129]
Preparation of nitazoxanide-loaded Lf NPs against SARS-CoV-2	*In vitro*	- Synthesis of nitazoxanide-Lf NPs with an average size of 353 ± 17 nm and a zeta potential of −13.9 ± 2.4 mV. - Enhanced Lf activity compared to its free form, supported by an improvement in the IC_50_ value from 2.273 μM to 1.438 μM.	[130]
Antifungal			
Development nanofiber membranes loaded with bLf to display antifungal activity against *Aspergillus nidulans*	*In vitro*	- The membranes had an overall porosity of around 80% and smooth, nondefective fibers with mean diameters ranging from 717 ± 197 to 495 ± 127 nm. - The presence of bLf decreases the hydrophobicity of the PLLA membranes. - Human fibroblasts were not cytotoxically affected by the bLf–PLLA membranes that were created; interestingly, after 24 h of indirect contact, the 20-weight-percent bLf–PLLA membrane was even capable of inducing cell growth.	[103]
Development of bLf-loaded PMLs as fungicidal agents	*In vitro*	- PMLs have been produced with gold NPs and manganese ferrite, functionalized with either octadecanethiol or 11-mercaptoundecanoic acid, and then loaded with bLf. - PMLs were formed when both plasmonic and magnetic NPs were enclosed in DPPC and Egg-PC liposomes. - PMLs loaded with bLf are around 200 nm in size, exhibit a positive zeta potential, and remain stable for at least five days. - Because PMLs are non-cytotoxic and retain their antifungal function, they exhibit encouraging potential for delivering bLf into yeast cells.	[122]
Development of alginate-enclosed chitosan–calcium phosphate-loaded Fe-Bovine Lf nanocapsules	*In vitro* *In vivo*	- The purpose of this study was to examine the anticandidal properties of Fe-bLf nanocapsules loaded with chitosan–calcium phosphate and encapsulated in alginate/EUDRAGIT R© S 100. - With an MIC value of 500 μg/mL, nanocapsules demonstrated strong antibacterial action against *C. albicans.* - Nanocapsules’ effectiveness in animal models was shown by an *in vivo* anticandidiasis investigation. Nanocapsules improved the epithelial cells’ ability to naturally destroy *C. albicans.*	[131]

## 4. Agriculture Applications of Lf NPs

The utilization of natural components in the field of agriculture, especially for current food packaging and food preservation applications, has significantly increased [85]. This is mostly attributed to the latest research on commercially employed chemical additives, which are known to cause several negative side effects, including carcinogenic effects and allergies, on consumer health [132].

To address this, researchers are using Lf as a natural additive in food storage and protection applications. Lately, many studies have been conducted in this field to enhance the shelf life of meat, fish, and fruits [133,134,135].

Being a less-toxic, biodegradable, and biocompatible alternative, Lf has also been employed in either its NP form or as a coating material for the development of reinforced preservation systems [136,137]. The main reason behind this is the superior antimicrobial activity of Lf, which effectively inhibits the proliferation of a wide array of pathogens to extend shelf life, prevent food spoilage, and increase crop yields.

### 4.1. Food Packaging

One of the main focuses of NP employment in the food industry is on the packaging systems. NPs are used to enhance the mechanical, barrier, and thermal properties of matrices, which paves the way for the production of strengthened food packaging materials [138]. However, their applicability is hindered by toxicity concerns, regarded as one of the main drawbacks associated with nanomaterials [139].

Hence, protein-based NPs emerged with their sustainable, renewable, and biodegradable nature to replace commercially used NPs [140]. Also, their relative abundance, oxygen barrier ability, film-formation capacity, and nutritional value further support their use in this area [141]. Lf, as an antimicrobial milk glycoprotein, possesses great potential for use in these applications. For example, it can be integrated into packaging films, either alone or combined with other proteins, to enhance the antibacterial activity and physicochemical properties of the final products [133,142].

Khezerlou et al. synthesized Lf-included NPs (chromium-based metal–organic frameworks) to develop gelatin–κ-carrageenan films as packaging materials to overcome limitations associated with perishable foods. The incorporation of Lf enhanced the functional properties of films, as evidenced by decreases from 105% to 70.8%, 61% to 34.63%, and 2.46 to 2.19 × 10^−11^ g·m/m^2^·s in swelling index, water solubility, and water permeability, respectively. Also, at increasing concentrations of Lf, antibacterial effects were observed against *S. aureus* and *E. coli* with inhibition zones of 20.2 and 19.7 mm. In packaging assays over 6 days, at 50% humidity and 25 °C, softening and mold growth on strawberries was observed with uncoated films, while the addition of Lf prevented these side effects and maintained the freshness [143].

Similarly, Tavassoli et al. developed bioactive packaging materials that contain Lf-encapsulated silver metal–organic framework NPs. The incorporation of these NPs improved the tensile strength, from 41.1 ± 2.4 MPa to 56.1 ± 3.2 MPa, and thermal stability while decreasing water vapor and oxygen permeability. In addition, bactericidal assays showed these films effectively inhibited the growth of both Gram-positive and Gram-negative bacteria, *S. aureus* and *E. coli*, with a maximum ZOI of 20.1 ± 3.2 mm and 19.8 ± 5.2 mm, in comparison to the films alone, 10.8 ± 6.1 and 10.6 ± 1.5 mm. Researchers examined the effect of Lf concentration (0.5, 1, and 2%) on the storage of fresh apples at 25 °C and 50% relative humidity for 7 days. Samples containing 2% Lf effectively extended the shelf life of apples and resulted in minimum visible changes, while their counterparts led to softness, dehydration, and change in color. Also, these films exhibited strong UV-protective properties, which indicated their further applicability in the protection of materials that are sensitive to photodegradation [144].

### 4.2. Food Preservation

Multiple studies in the current literature highlight the use of nanomaterials, especially inorganic ones, for effective food preservation [145,146]. Specifically, metallic and metal oxide NPs (including silver, zinc oxide, and titanium dioxide) have gained attention in these systems with their strong antimicrobial activity [138,147].

However, similar to food packaging, there are rising concerns about the toxicity potential of these NPs due to their inherent non-biodegradability [148,149]. Considering these, researchers are utilizing organic-based food contact materials, like proteins, to create non-toxic and novel approaches. Lf stands out in this aspect owing to its strong antibacterial efficiency and previously demonstrated strong food preservation capability [150,151,152]. Its ease of functionalization and combined effects with NPs further support its use in this field and underscore its promising potential in future studies.

A recent study involved the combined use of Lf NPs with other biomolecules, chitosan and gellan gum, to extend the shelf life of fresh strawberries. It has been observed that coating with NPs significantly enhanced the physicochemical properties of strawberries in comparison to their uncoated counterparts, which showed shrinkage, mold, and darker color following 96 h of storage at 25 °C. These ternary NPs exhibited noteworthy antibacterial activity against *S. aureus* (at a concentration of 10^3^ CFU), with MIC values as low as 0.0117 mg/mL. Also, researchers conducted aerobic bacteria counts *in natura* to assess overall fruit quality. After 144 h of storage, bacterial concentration increased from 2.47 Log CFU/mL to 4.2 Log CFU/mL for the untreated (control) sample, while it remained at 3.74 Log CFU/mL for NP-treated fruits [153].

Similarly, Duarte et al. focused on producing natural preservatives to improve the shelf life of strawberries. Aiming to exploit the combined antimicrobial effect of bovine Lf and chitosan, researchers synthesized Lf–chitosan–sodium tripolyphosphate NPs. These NPs demonstrated potent antibacterial activity against *S. aureus* (with an MIC value of 0.0370 mg/mL) indicating a three times lower concentration than the pure materials. Further, coated strawberries were stored at 25 °C and 50% relative humidity for 6 days. The results revealed darker colors, shrinking, and water loss in uncoated samples, while NP coating led to delays in food ripening and degradation [136].

Lf is a highly effective antimicrobial biomolecule with significant potential in food packaging and preservation applications. Both Lf and Lf-derived peptides, particularly lactoferricin, are widely used and preferred biomaterials in agricultural applications, as they enhance antioxidant and antibacterial activity while improving the mechanical and thermal properties of food packages [85,154]. Furthermore, there is growing interest in utilizing various antimicrobial NPs and nanocomposites for food preservation applications to enhance similar attributes in food packaging [155]. However, their combined application in agriculture remains quite limited, despite the proven efficiency of their individual use in these applications over recent years. Given the recent focus on Lf-based NPs in the literature, the lack of significant development in this area is not surprising. Significant attention has been given to the antimicrobial properties of Lf-based nanomaterials in other fields, particularly in antibacterial drug delivery applications. Even this sub-field requires further advancements to fully demonstrate the potential of Lf-based nanomaterials. Given the current state of research, there is a pressing need for systematic studies on Lf NPs, focusing on their safety, efficacy, and feasibility for agricultural applications.

## 5. Toxicity

Lf is a primary antimicrobial component found abundantly in milk and colostrum [156], making it a preferred, safe agent for various therapeutic applications. Depending on its source, Lf may vary in molecular structure due to differences in amino acid sequences and the structure of glycans [7]. Consequently, its effectiveness in specific applications can vary, as demonstrated by previous studies [157,158]. Therefore, selecting the appropriate type of Lf is crucial to prevent any undesired outcomes in the application.

bLf represents the largest scale in industrial production due to its abundant availability, making it suitable for therapeutic and supplementary uses [159]. Consequently, bLf has received approval from the United States Food and Drug Administration and is recognized as a safe dietary supplement by the European Food Safety Authority [78]. These approvals place Lf in a reassuring position regarding toxicity concerns. However, it remains necessary to evaluate optimal dosing approaches to prevent any toxic outcomes.

One study investigated bovine Lf’s optimum dose of administration for preterm infants [160]. Thirty-one patients were divided into three groups, receiving 100, 200, and 300 mg/kg of daily bovine Lf for 10 months. At the end of the supplementation, none of the patients showed any Lf-based adverse effects, showing notable tolerance at the dose of 300 mg/kg. In the past few years, studies that involved investigating the effects of bovine Lf supplementation used similar doses during the experimentation. One study used 200 mg/kg daily bovine Lf to investigate its effect against sepsis and neurodevelopment impairment in infants [161]. Although the trial did not show any effect, the supplementation did not create any adverse effects. Another study used 200 mg to 1000 mg daily bovine Lf for COVID-19 patients, showing reduced symptoms and no adverse effects [162].

The latest experiments on Lf toxicity and clinical trials using naturally sourced Lf primarily focus on determining the optimal dose for administration. To investigate Lf toxicity further, recombinant synthesis of Lf can be centered since it holds significant potential for future research on Lf.

Recombinant Lfs (rLfs) holds promise as an alternative source for advancing Lf-based NP applications. It exhibits a great alternative to obtain high amounts of Lf for conducting wide-scale applications and possible productization. This is why recombinant technology is commonly preferred for human Lfs, given the challenges of obtaining it from natural sources for large-scale production. Still, given the current uncertainties regarding safety conditions, rLfs face significant challenges in their application. One of the main reasons behind this problem is the alterations in the biological activity of Lfs due to the recombinant synthesis, given the possible variables affecting the glycosylation patterns including monosaccharide composition and connection with glycan structures [163,164]. As a result, current research increasingly focuses on assessing the toxicological characteristics of recombinant human Lfs for future applications.

One of the most recent developments in the investigation of rLf was mediated with human Lf (hLf) which is synthesized from glycoengineered yeast, *Komatagaella phaffii*. On this issue, a workshop report has been published that discussed the roadmap to achieve a safety standard for hLf [165]. According to the report, certain safety factors need to be evaluated: determination of any potential immunotoxicity from rLf ingestion, potential effects of rLf in iron homeostasis, any trace of alloimmunization, and the pathway that rLf potentially goes through during the digestion.

Recently, recombinant hLf was tested in an *in vivo* study to determine the dose range for further research [166]. Rats were separated into three different groups, each receiving a different dose of recombinant hLf for 14 days: 200, 1000, and 2000 mg/kg. A fourth control group consumed 2000 mg/kg of bovine Lf daily. The toxicological results indicated that rats demonstrated high tolerance to 2000 mg/kg daily consumption of recombinant hLf, allowing a reference dose for further studies. Subsequently, the same research group tested this recombinant hLf for potential immunogenicity, comparing it to bovine Lf in a randomized, double-blind, controlled trial [167]. The trial consisted of three main groups (66 healthy individuals), whereas two groups consumed human rLf, taking 0.34 and 3.4 g per day, and the third group consumed 3.4 g of bLf per day. On day 56, it was shown that intake of human rLf did not induce any alloimmunization or increase the levels of hLf-antibodies.

Given Lf’s features and the glycan structure variations that can significantly impact its activity, similar safety concerns must be thoroughly investigated to include rLfs in products intended for supplements or therapeutic applications. Recombinant Lf production could provide a strong foundation for future Lf-based NP applications and be a basis for upcoming clinical trials. Further research could support the safe use of rLfs in NP-based applications. However, certain types of NPs carry higher health risks and safety concerns compared to the Lf protein.

**Figure 6:**
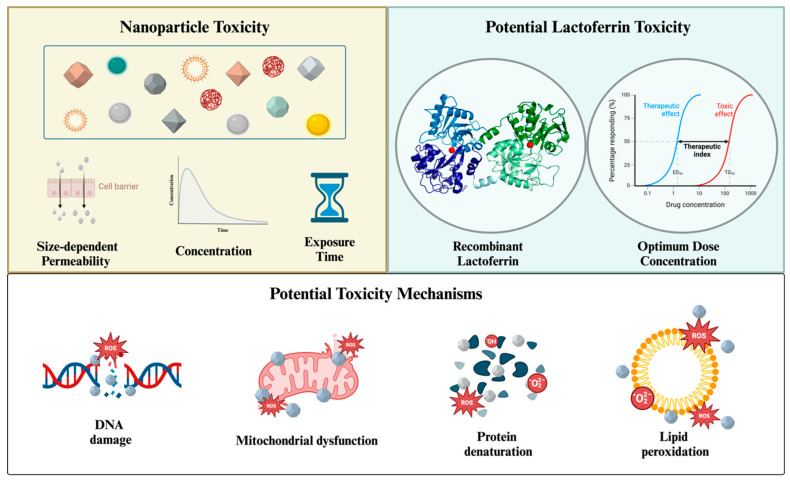
Potential toxicity of NPs and Lf. The toxicity capacity of NPs depends on not only the type of the NPs, but also the exposure time, concentration, and physicochemical properties, specifically their size. High-concentration and recombinantly produced Lf can cause potential adverse effects on the immune system. As a result, similar to most NP-based applications, the following toxicity mechanisms can be observed in Lf-based NP applications: ROS-mediated DNA damage, protein denaturation, lipid peroxidation, and mitochondrial dysfunction [28,171].

Regarding the characteristics of the NPs, particular types have been viewed as having high toxicity potential (Figure 6). With their small size and high reactivity, NPs can exhibit high toxicity depending on their concentration, exposure time, and route of administration [168]. One of the main mechanisms behind NPs toxicity is ROS synthesis. NPs can increase the oxidative stress levels that lead to DNA damage, protein denaturation, lipid peroxidation, and/or mitochondrial dysfunction [28,169,170].

In this context, the potential toxicity associated with the type of NP used in combination with Lf should be thoroughly evaluated. Many metal and metal oxide NPs exhibit significant toxicity potential, which is influenced by their physicochemical properties, including size, shape, surface charge, uptake efficiency, and aggregation potential through stability [172]. Still, these particles have significant activity and potential in many applications that involve NP research, including those involving Lf. Certain types of metal NPs, such as iron oxide and silver, exhibit great potential and efficiency in Lf-based applications [71,173]. Given the enhanced results of Lf-based metal NPs in the discussed applications, various factors should be considered to improve the efficiency-to-toxicity ratio while maintaining the non-toxic nature of the application. During the synthesis of Lf-based NPs, changes in physicochemical properties should be closely monitored, as they directly influence the toxicity potential of the particles [174]. The addition of Lf to metal NPs not only significantly affects particle size but also influences surface charge and solubility, thereby impacting their toxicity potential [175].

One factor significantly influenced by the addition of Lf is the stability of the particles. Metal NPs exhibit lower toxicity potential when they demonstrate high stability, reducing their tendency to aggregate in the environment [176]. Various approaches have been developed to ensure the stability of NPs during synthesis processes. Surface modification is among the most common methods for preserving the stability of particles in different environments. Protein coatings are notable options for enhancing stability, as they can reduce particle aggregation with other proteins and promote electrostatic stabilization by influencing charge density [177]. Lf as a coating material might similarly enhance the stability of NPs, alleviating safety concerns while improving application efficiency. However, research directly investigating the relationship between Lf coatings and NP stability remains significantly limited. Experiments focusing on the changes in physicochemical properties of NPs through Lf encapsulation and coatings could reveal new strategies to ensure the stability of Lf-based NPs in various applications.

In addition to physicochemical aspects, general applications for reducing the toxicity capacity of metal NPs should also be considered. Surface modification or encapsulation of Lf can greatly reduce the toxicity effects of metal NPs. A study highlighted the enhanced adjuvanticity of Lf-absorbed silver NPs, which exhibited increased and prolonged IgG levels [116]. It is suggested that enhanced internalization and transport of antigens facilitate the desired immune response. Moreover, it was emphasized that Lf absorption mitigates the toxicity potential of silver NPs by reducing ROS levels compared to administrating silver NPs alone *in vitro*. While higher concentrations of silver NPs reduced cell viability by 50% (at the highest concentration of 120 pM), Lf absorption significantly minimized this effect, maintaining a 25% reduction in viability over high doses. Another study demonstrated that surface modification with Lfs reduces non-specific cellular internalization of iron oxide NPs [178]. The modified NPs induced ligand–receptor interactions with high affinity with surface modifications, reducing the possibility of random endocytosis during applications.

Although surface modification of NPs through coating can increase stability and reduce non-specific internalization, it may also create pathways that induce undesired toxicity outcomes. In drug delivery applications, various plasma proteins can interact with different types of NPs through electrostatic, hydrophobic, or polar interactions [179]. Under certain conditions, positively charged Lf can alter the charge density of conjugated NPs, affecting their ability to initiate electrostatic interactions. Furthermore, given Lf’s influence on the immune system, its presence can induce or suppress immune responses, adding another factor to consider during application. Additionally, NPs tend to form protein coronas after administration, which affects their distribution and circulation time in the body [180]. Surface coating of NPs can reduce protein corona formation, enhancing NP-based drug delivery applications while mitigating toxicity-related adverse effects. Lf is known for its multifunctional characteristics and its ability to interact with various types of molecules. Therefore, the impact of Lf coating on protein corona formation should be investigated, as it may contribute to toxicity rather than enhancing activity. Given the ongoing toxicity concerns surrounding rLfs, employing recombinant technology in this area before optimizing naturally occurring Lfs presents significant challenges. The use of rLfs may amplify undesired effects on the immune system during administration.

The use of various coatings and surface modification methods to reduce the toxicity of metal NPs has been widely explored, particularly in drug delivery applications [181]. From this perspective, Lf coating might yield similar results in metal NPs; however, a robust research foundation and targeted experiments are still necessary to draw such a conclusion.

We have discussed some types of NPs that have been used with Lf in various applications. Excluding the organic-based structures, metal-based NPs have been commonly used in these applications. Many metallic NPs, such as silver, gold, and iron oxide, have shown high efficiency in delivering various natural products [182]. Moreover, these NPs exhibit significant potential in future clinical trials, such as in antimicrobial therapy, antitumor application, additive material for bone healing, and so on [183]. These applications are mostly common with the biological activities of Lf, indicating the possibility of future therapies based on Lf-based NP applications. It needs to be highlighted that most of the common metal-based NPs are known to have a high potential for inducing oxidative stress, depending on their physicochemical properties [184].

## 6. Conclusions

Thanks to its multifunctional properties, Lf exhibits significant applicability across various fields. Nanotechnology is an emerging field that offers promising approaches in various applications. Considering the characteristics of Lf, there is a great possibility that Lf will have a major interest in NP technology. Lf demonstrates strong antibacterial properties, immunomodulatory activity, and potential use in agricultural applications with minimal adverse effects. Moreover, due to the rising demand for alternative therapeutic approaches and carriers, Lf’s properties can significantly be utilized for developing drug delivery strategies, particularly in NP technology. Lf can not only be conjugated and modified on NPs for various therapeutic applications but can also function as NPs itself, showcasing its versatility. As Lf receptors are present in key human tissues, particularly the BBB, Lf has the potential to drive the development of novel delivery strategies that address several existing challenges in the field.

Still, despite the multifunctionality of the protein, certain areas of Lf-based NP applications remain quite limited. One of the most overlooked areas where Lf holds significant potential is agriculture. As covered in the agriculture section, only a limited number of studies have been published in recent years where Lf is combined with NP technology in agriculture-based applications. Interestingly, the current literature indicates that both Lf and NPs have been individually utilized in agricultural applications with a wide-ranging approach. It is somewhat surprising that these two approaches are not combined more frequently. In the future, with sufficient research support, Lf-based NP applications could be utilized in various agricultural areas, particularly in food preservation.

Another deficient part that holds great potential is the NP-based delivery of Lf. Beyond its role as a supportive molecule, the significance of Lf’s biological activity has been extensively discussed in recent years. Lf exerts its activity in various parts of the body, with effects that can vary depending on the specific location. With the necessary optimizations, site-directed delivery of Lf could have a significant impact on various therapeutic applications, such as anticancer, anti-inflammatory, and neuroprotective therapies. However, the range of these applications in recent years remains quite limited, despite the proven efficacy of Lf in these areas. Various types of NPs have been demonstrated to be excellent carriers for therapeutic agents, highlighting them for advanced delivery methodologies. Conducting further research on NP-based Lf delivery could yield significant outcomes in numerous therapies that require improvements and novel alternatives.

Toxicity concerns remain a significant challenge that requires careful consideration for the broad application of Lf in NP technology. While Lf coating offers a promising alternative to reduce the toxicity of NPs, its effects on immune system interactions and molecular interactions with various proteins remain unclear. Research is needed to understand how Lf coating influences the physicochemical properties and stability of NPs to support their wide-scale applicability. Additionally, comprehensive reviews on the different types and sources of Lf are needed in the literature to identify the most suitable type for NP-based applications. Further research conducted to test and compare different sources of Lf, including recombinantly synthesized variants, could help identify the most suitable option to address toxicity concerns and enhance application efficiency.

In summary, Lf demonstrates key characteristics that position it as a potential therapeutic agent in NP-based nanomedicine applications. The current literature highlights several significant and promising sub-areas of Lf-based NP applications. With sufficient research, additional sub-areas are likely to emerge as Lf is further integrated into NP technology. Future research could not only expand the range of Lf applications but also enhance existing NP applications in various emerging fields.

## Data Availability

Not applicable.
